# ASO Author Reflections: Evolution of Gastric Cancer Care at Tata Memorial Centre—Best of the East and the West

**DOI:** 10.1245/s10434-024-15929-y

**Published:** 2024-08-04

**Authors:** Shailesh V. Shrikhande, Swati Batra, Vikram A. Chaudhari, Manish S. Bhandare

**Affiliations:** Department of Surgical Oncology, Gastrointestinal and HPB Service, Tata Memorial Centre, Homi Bhabha National Institute, Parel, Mumbai, India

## PAST

In parallel with a uniform global rise in the incidence of gastric cancer (GC), the past two decades have witnessed significant advancements in GC management, with improvement of lymphadenectomy techniques, adjuvant/neo-adjuvant therapies, and use of minimal access surgery.^1–3^ However, the outcomes for resected advanced gastric cancers (AGCs) are less reported.

## PRESENT

The GC care offered at Tata Memorial Centre reflects a blend of strategies from East and West (i.e., consistent adoption of D2 dissection with a predominant perioperative chemotherapy approach). This study highlights the evolution of AGC management with a comparison of outcomes during different periods of the study.^4^ More than 90% of the included patients had AGC. In a 17-year span, the study observed significant expansion of surgical volume. Extended indications for curative resection (multivisceral resections and cytoreduction with hyperthermic intraperitoneal chemotherapy [HIPEC]) in AGCs were used in the later study period. Overall, gradual and sustained improvement in surgical quality indicators were observed, with consistently low morbidity and mortality rates and an excellent median survival of 4 years.

## FUTURE

The encouraging outcomes observed are clearly a reflection of increasing experience and a result of the “volume/centre effect” from a dedicated gastric cancer care unit. These results strongly indicate the need for the development of specialized centers managing GC.

Further refinements in AGC management may involve inclusion of extended lymphadenectomy in specific scenarios and establishment of stringent criteria for offering peritoneal directed therapies and extended resections. An ongoing ELANCe trial (a phase III Randomized Controlled Trial Comparing D2 Versus D3 Lymphadenectomy) at our center should provide important insights on the role of extended lymphadenectomy in AGCs.^5^
